# Tetra­aqua­bis(*N*-phenyl­sulfonyl-l-leucinato)cadmium(II) dihydrate

**DOI:** 10.1107/S1600536809000683

**Published:** 2009-01-10

**Authors:** Pei-Guo Guan

**Affiliations:** a Department of Sport, Weifang University, Weifang 261061, People’s Republic of China

## Abstract

In the title compound, [Cd(C_12_H_16_NO_4_S)_2_(H_2_O)]·2H_2_O, the Cd atom is located on a twofold rotation axis and a distorted CdO_8_ dodeca­hedral arrangement arises from the coordination of the two chelating ligands and four water mol­ecules. A network of N—H⋯O, O—H⋯O and C—H⋯O hydrogen bonds help to establish the crystal packing. Both coordinated and uncoordinated water molecules are disordered with an approximate half-occupation for each of the water molecules.

## Related literature

For background to the design and synthesis of metal complexes, see: Zhang *et al.* (2007 ▶).
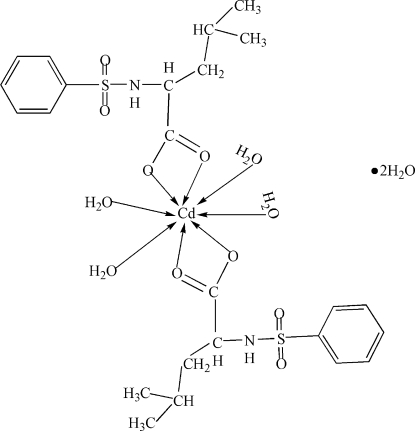


## Experimental

### 

#### Crystal data

[Cd(C_12_H_16_NO_4_S)_2_(H_2_O)]·2H_2_O
*M* *_r_* = 725.10
Orthorhombic, 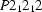

*a* = 17.733 (2) Å
*b* = 17.2930 (19) Å
*c* = 5.6051 (11) Å
*V* = 1718.9 (4) Å^3^
*Z* = 2
Mo *K*α radiation
μ = 0.81 mm^−1^
*T* = 298 (2) K
0.50 × 0.40 × 0.36 mm

#### Data collection

Bruker SMART CCD area-detector diffractometer
Absorption correction: multi-scan (*SADABS*; Sheldrick, 1996 ▶) *T*_min_ = 0.687, *T*_max_ = 0.759
9050 measured reflections
3033 independent reflections
1954 reflections with *I* > 2σ(*I*)
*R*_int_ = 0.051

#### Refinement

*R*[*F* ^2^ > 2σ(*F* ^2^)] = 0.068
*wR*(*F* ^2^) = 0.214
*S* = 1.03
3033 reflections
207 parameters
H-atom parameters constrained
Δρ_max_ = 0.70 e Å^−3^
Δρ_min_ = −0.56 e Å^−3^
Absolute structure: Flack (1983 ▶), 1247 Friedel pairs
Flack parameter: 0.00 (8)

### 

Data collection: *SMART* (Bruker, 1997 ▶); cell refinement: *SAINT* (Bruker, 1997 ▶); data reduction: *SAINT*; program(s) used to solve structure: *SHELXS97* (Sheldrick, 2008 ▶); program(s) used to refine structure: *SHELXL97* (Sheldrick, 2008 ▶); molecular graphics: *SHELXTL* (Sheldrick, 2008 ▶); software used to prepare material for publication: *SHELXTL*.

## Supplementary Material

Crystal structure: contains datablocks global, I. DOI: 10.1107/S1600536809000683/at2702sup1.cif

Structure factors: contains datablocks I. DOI: 10.1107/S1600536809000683/at2702Isup2.hkl

Additional supplementary materials:  crystallographic information; 3D view; checkCIF report

## Figures and Tables

**Table 1:** Hydrogen-bond geometry (Å, °)

*D*—H⋯*A*	*D*—H	H⋯*A*	*D*⋯*A*	*D*—H⋯*A*
N1—H1⋯O1	0.90	2.29	2.776 (11)	113
N1—H1⋯O2^i^	0.90	2.35	3.121 (12)	143
O5—H5*E*⋯O1^ii^	0.85	1.85	2.639 (19)	154
O5—H5*F*⋯O1^iii^	0.85	1.79	2.605 (18)	161
O7—H7*C*⋯O3^iv^	0.85	2.20	2.99 (2)	155
O7—H7*D*⋯O4^v^	0.85	2.22	3.00 (2)	152
C2—H2⋯O3	0.98	2.46	2.903 (13)	107
C2—H2⋯O4^ii^	0.98	2.58	3.457 (13)	149
C12—H12⋯O3	0.93	2.52	2.871 (14)	102

Symmetry codes: (i) 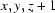; (ii) 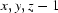; (iii) 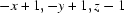; (iv) 
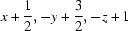
; (v) 
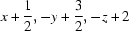
.

**Fig. 1.**
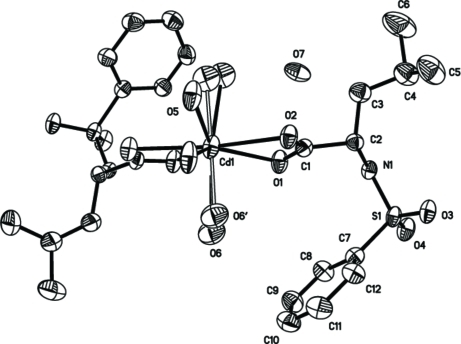
The complex molecule, with 30% probabiility ellipsoids.

## supplementary crystallographic information

### Comment

During the last decade, the design and synthesis of metal complexes have attracted considerable attention due to their potential uses as biological activities (Zhang *et al.*, 2007). The synthesis and structure of the title compound (I) is reported.

In the title compound, the Cd atom is located on an inversion center. Two O-bidentate ligands and four water molecules are attached to the cadmium atom, resulting in a distorted CdO_8_ triangluar dodecahedral arrangement (Fig. 1). The identical S1═O3 [1.407 (7) Å], S1═O4 [1.430 (8) Å] and C1═O2 [1.235 (13) Å] bonds lengths imply double-bond character. The dihedral angle between the two benzene ring mean planes (C7—C12 and C7A—C12A) is 58.2 °.

Two molecules of water complete the structure of (I) and a network of hydrogen bonds helps to establish the crystal packing (Table 1).

### Experimental

1 mmol of cadmium chloride was added to a solution of 2-phenylsulfonyl chloride-L-leucine (2 mmol) in 10 ml of CH_3_OH/H_2_O (*v*/*v* 1:1). The mixture was continuously stirred for 4 h at refluxing temperature, evaporating some methanol, then, upon cooling, the solid product was collected by filtration and dried *in vacuo* (yield 69%). Clear blocks of (I) were obtained by evaporation from a methanol solution after a week.

### Refinement

The water H atoms were located in a difference map and refined as riding in their as-found relative positions with *U*_iso_(H) = 1.2*U*_eq_(O). Other H atoms were placed geometrically (C—H = 0.93–0.98 Å, O—H = 0.82 Å, N—H = 0.90 Å) and refined as riding with *U*_iso_(H) = 1.2*U*_eq_(C,N) or 1.5*U*_eq_(C,O).

### Crystal data

**Table:**

[Cd(C_12_H_16_NO_4_S)_2_(H_2_O)]·2H_2_O	*F*(000) = 748
*M**_r_* = 725.10	*D*_x_ = 1.401 Mg m^−^^3^
Orthorhombic, *P*2_1_2_1_2	Mo *K*α radiation, λ = 0.71073 Å
Hall symbol: P 2 2ab	Cell parameters from 2267 reflections
*a* = 17.733 (2) Å	θ = 2.3–19.6°
*b* = 17.2930 (19) Å	µ = 0.81 mm^−^^1^
*c* = 5.6051 (11) Å	*T* = 298 K
*V* = 1718.9 (4) Å^3^	Block, colourless
*Z* = 2	0.50 × 0.40 × 0.36 mm

### Data collection

**Table:**

Bruker SMART CCD area-detector diffractometer	3033 independent reflections
Radiation source: fine-focus sealed tube	1954 reflections with *I* > 2σ(*I*)
graphite	*R*_int_ = 0.051
φ and ω scans	θ_max_ = 25.0°, θ_min_ = 1.6°
Absorption correction: multi-scan (*SADABS*; Sheldrick, 1996)	*h* = −21→19
*T*_min_ = 0.687, *T*_max_ = 0.759	*k* = −18→20
9050 measured reflections	*l* = −6→6

### Refinement

**Table:**

Refinement on *F*^2^	Secondary atom site location: difference Fourier map
Least-squares matrix: full	Hydrogen site location: inferred from neighbouring sites
*R*[*F*^2^ > 2σ(*F*^2^)] = 0.068	H-atom parameters constrained
*wR*(*F*^2^) = 0.214	*w* = 1/[σ^2^(*F*_o_^2^) + (0.1333*P*)^2^ + 0.5509*P*] where *P* = (*F*_o_^2^ + 2*F*_c_^2^)/3
*S* = 1.03	(Δ/σ)_max_ = 0.001
3033 reflections	Δρ_max_ = 0.70 e Å^−^^3^
207 parameters	Δρ_min_ = −0.56 e Å^−^^3^
0 restraints	Absolute structure: Flack (1983), 1247 Freidel pairs
Primary atom site location: structure-invariant direct methods	Flack parameter: 0.00 (8)

### Special details

**Table:**

Geometry. All e.s.d.'s (except the e.s.d. in the dihedral angle between two l.s. planes) are estimated using the full covariance matrix. The cell e.s.d.'s are taken into account individually in the estimation of e.s.d.'s in distances, angles and torsion angles; correlations between e.s.d.'s in cell parameters are only used when they are defined by crystal symmetry. An approximate (isotropic) treatment of cell e.s.d.'s is used for estimating e.s.d.'s involving l.s. planes.
Refinement. Refinement of *F*^2^ against ALL reflections. The weighted *R*-factor *wR* and goodness of fit *S* are based on *F*^2^, conventional *R*-factors *R* are based on *F*, with *F* set to zero for negative *F*^2^. The threshold expression of *F*^2^ > σ(*F*^2^) is used only for calculating *R*-factors(gt) *etc*. and is not relevant to the choice of reflections for refinement. *R*-factors based on *F*^2^ are statistically about twice as large as those based on *F*, and *R*- factors based on ALL data will be even larger.

### Fractional atomic coordinates and isotropic or equivalent isotropic displacement parameters (Å^2^)

**Table:**

	*x*	*y*	*z*	*U*_iso_*/*U*_eq_	Occ. (<1)
Cd1	0.5000	0.5000	0.05213 (18)	0.0754 (4)	
N1	0.3790 (4)	0.7120 (5)	0.5321 (16)	0.067 (2)	
H1	0.3992	0.6707	0.6057	0.081*	
O1	0.4512 (4)	0.5829 (4)	0.3465 (14)	0.079 (2)	
O2	0.4394 (4)	0.6239 (4)	−0.0210 (14)	0.077 (2)	
O3	0.2573 (4)	0.7608 (4)	0.4174 (14)	0.080 (2)	
O4	0.2793 (4)	0.7154 (5)	0.8283 (13)	0.085 (2)	
O5	0.5451 (10)	0.5261 (9)	−0.333 (3)	0.099 (5)	0.50
H5E	0.5163	0.5567	−0.4087	0.119*	0.50
H5F	0.5517	0.4850	−0.4128	0.119*	0.50
O6	0.379 (2)	0.446 (3)	0.075 (15)	0.110 (12)	0.57 (13)
H6E	0.3791	0.3977	0.0942	0.132*	0.57 (13)
H6F	0.3768	0.4554	−0.0740	0.132*	0.57 (13)
O6'	0.396 (3)	0.428 (3)	0.191 (19)	0.110 (16)	0.43 (13)
H6'C	0.3941	0.3821	0.1354	0.132*	0.43 (13)
H6'B	0.4170	0.4296	0.3270	0.132*	0.43 (13)
O7	0.6587 (11)	0.6808 (11)	0.976 (4)	0.128 (7)	0.50
H7C	0.6778	0.6880	0.8389	0.153*	0.50
H7D	0.6855	0.7054	1.0766	0.153*	0.50
S1	0.29018 (13)	0.70772 (14)	0.5767 (5)	0.0649 (6)	
C1	0.4350 (6)	0.6338 (6)	0.197 (2)	0.068 (3)	
C2	0.4090 (6)	0.7135 (6)	0.2887 (19)	0.069 (3)	
H2	0.3714	0.7354	0.1798	0.083*	
C3	0.4805 (6)	0.7630 (7)	0.285 (2)	0.085 (3)	
H3A	0.5139	0.7441	0.4090	0.102*	
H3B	0.5056	0.7546	0.1336	0.102*	
C4	0.4717 (8)	0.8475 (8)	0.319 (3)	0.101 (4)	
H4	0.4474	0.8621	0.4690	0.122*	
C5	0.4321 (9)	0.8787 (9)	0.095 (4)	0.129 (6)	
H5A	0.4440	0.8463	−0.0392	0.194*	
H5B	0.4491	0.9305	0.0636	0.194*	
H5C	0.3786	0.8789	0.1199	0.194*	
C6	0.5520 (11)	0.8810 (9)	0.286 (4)	0.151 (8)	
H6A	0.5840	0.8629	0.4117	0.227*	
H6B	0.5497	0.9365	0.2897	0.227*	
H6C	0.5719	0.8646	0.1346	0.227*	
C7	0.2568 (6)	0.6164 (6)	0.498 (2)	0.074 (3)	
C8	0.2721 (7)	0.5536 (7)	0.651 (2)	0.083 (3)	
H8	0.2995	0.5616	0.7905	0.099*	
C9	0.2469 (7)	0.4809 (7)	0.596 (3)	0.093 (4)	
H9	0.2571	0.4398	0.6977	0.111*	
C10	0.2057 (8)	0.4681 (8)	0.384 (3)	0.094 (4)	
H10	0.1882	0.4189	0.3462	0.113*	
C11	0.1923 (7)	0.5283 (7)	0.239 (3)	0.093 (4)	
H11	0.1650	0.5202	0.1000	0.112*	
C12	0.2186 (7)	0.6048 (7)	0.292 (2)	0.083 (3)	
H12	0.2096	0.6454	0.1871	0.099*	

### Atomic displacement parameters (Å^2^)

**Table:**

	*U*^11^	*U*^22^	*U*^33^	*U*^12^	*U*^13^	*U*^23^
Cd1	0.0923 (8)	0.0840 (7)	0.0497 (6)	0.0321 (6)	0.000	0.000
N1	0.075 (5)	0.069 (4)	0.058 (5)	0.015 (4)	−0.007 (4)	−0.005 (4)
O1	0.095 (5)	0.077 (4)	0.064 (5)	0.024 (4)	−0.006 (4)	0.004 (4)
O2	0.090 (5)	0.079 (4)	0.062 (5)	0.026 (4)	0.002 (4)	0.000 (4)
O3	0.084 (4)	0.080 (5)	0.076 (5)	0.030 (4)	−0.004 (4)	0.003 (4)
O4	0.099 (5)	0.102 (5)	0.056 (4)	0.030 (5)	0.010 (4)	−0.010 (4)
O5	0.149 (14)	0.083 (11)	0.066 (10)	0.054 (9)	0.002 (9)	0.006 (8)
O6	0.115 (16)	0.120 (16)	0.09 (3)	0.017 (12)	−0.012 (19)	0.005 (18)
O6'	0.11 (2)	0.12 (2)	0.09 (3)	0.017 (17)	−0.01 (2)	0.00 (3)
O7	0.133 (15)	0.133 (14)	0.117 (17)	−0.040 (12)	0.014 (13)	−0.052 (14)
S1	0.0736 (14)	0.0714 (14)	0.0498 (13)	0.0225 (12)	−0.0001 (13)	−0.0047 (13)
C1	0.074 (6)	0.071 (6)	0.058 (7)	0.015 (5)	−0.006 (5)	0.008 (6)
C2	0.077 (7)	0.071 (6)	0.060 (6)	0.009 (5)	−0.004 (5)	0.003 (5)
C3	0.087 (8)	0.088 (8)	0.080 (8)	0.003 (6)	−0.008 (6)	0.006 (6)
C4	0.102 (9)	0.103 (10)	0.099 (10)	−0.002 (7)	−0.018 (8)	0.005 (9)
C5	0.142 (13)	0.119 (11)	0.126 (15)	−0.002 (9)	−0.027 (13)	0.022 (12)
C6	0.147 (15)	0.137 (14)	0.17 (2)	−0.037 (12)	−0.029 (15)	0.013 (15)
C7	0.083 (6)	0.080 (6)	0.060 (8)	0.008 (5)	−0.001 (5)	0.003 (5)
C8	0.095 (8)	0.085 (8)	0.069 (8)	0.004 (6)	−0.008 (6)	0.002 (6)
C9	0.104 (8)	0.089 (9)	0.085 (9)	0.002 (6)	−0.004 (7)	0.011 (7)
C10	0.106 (9)	0.091 (8)	0.086 (10)	−0.008 (7)	0.003 (8)	0.003 (8)
C11	0.105 (9)	0.099 (10)	0.075 (9)	−0.006 (7)	−0.008 (7)	0.000 (7)
C12	0.096 (8)	0.086 (8)	0.067 (8)	−0.002 (6)	−0.003 (7)	0.003 (6)

### Geometric parameters (Å, °)

**Table:**

Cd1—O5^i^	2.343 (16)	S1—C7	1.742 (12)
Cd1—O5	2.343 (16)	C1—C2	1.543 (15)
Cd1—O6	2.35 (3)	C2—C3	1.531 (15)
Cd1—O6^i^	2.35 (3)	C2—H2	0.9800
Cd1—O1^i^	2.351 (7)	C3—C4	1.481 (17)
Cd1—O1	2.351 (7)	C3—H3A	0.9700
Cd1—O6'^i^	2.36 (4)	C3—H3B	0.9700
Cd1—O6'	2.36 (4)	C4—C5	1.54 (2)
Cd1—O2^i^	2.433 (7)	C4—C6	1.55 (2)
Cd1—O2	2.433 (7)	C4—H4	0.9800
Cd1—C1^i^	2.709 (11)	C5—H5A	0.9600
N1—C2	1.464 (14)	C5—H5B	0.9600
N1—S1	1.597 (8)	C5—H5C	0.9600
N1—H1	0.8999	C6—H6A	0.9600
O1—C1	1.249 (12)	C6—H6B	0.9600
O2—C1	1.235 (13)	C6—H6C	0.9600
O3—S1	1.407 (7)	C7—C12	1.356 (16)
O4—S1	1.430 (8)	C7—C8	1.409 (16)
O5—H5E	0.8500	C8—C9	1.371 (17)
O5—H5F	0.8500	C8—H8	0.9300
O6—H6E	0.8500	C9—C10	1.408 (19)
O6—H6F	0.8501	C9—H9	0.9300
O6—H6'C	1.1951	C10—C11	1.343 (16)
O6'—H6E	0.8094	C10—H10	0.9300
O6'—H6'C	0.8500	C11—C12	1.432 (17)
O6'—H6'B	0.8501	C11—H11	0.9300
O7—H7C	0.8500	C12—H12	0.9300
O7—H7D	0.8499		
			
O5^i^—Cd1—O5	46.1 (9)	H6E—O6—H6'C	15.9
O5^i^—Cd1—O6	70 (2)	H6F—O6—H6'C	117.1
O5—Cd1—O6	116 (2)	Cd1—O6'—H6E	114.2
O5^i^—Cd1—O6^i^	116 (2)	Cd1—O6'—H6'C	113.8
O5—Cd1—O6^i^	70 (2)	H6E—O6'—H6'C	30.9
O6—Cd1—O6^i^	174 (4)	Cd1—O6'—H6'B	86.3
O5^i^—Cd1—O1^i^	130.8 (4)	H6E—O6'—H6'B	141.9
O5—Cd1—O1^i^	129.6 (4)	H6'C—O6'—H6'B	112.3
O6—Cd1—O1^i^	93 (2)	H7C—O7—H7D	107.7
O6^i^—Cd1—O1^i^	82.2 (9)	O3—S1—O4	120.6 (5)
O5^i^—Cd1—O1	129.6 (4)	O3—S1—N1	106.2 (5)
O5—Cd1—O1	130.8 (4)	O4—S1—N1	106.4 (5)
O6—Cd1—O1	82.2 (9)	O3—S1—C7	106.9 (5)
O6^i^—Cd1—O1	93 (2)	O4—S1—C7	106.7 (5)
O1^i^—Cd1—O1	90.8 (4)	N1—S1—C7	109.7 (5)
O5^i^—Cd1—O6'^i^	132 (3)	O2—C1—O1	123.5 (10)
O5—Cd1—O6'^i^	86 (3)	O2—C1—C2	118.2 (10)
O6—Cd1—O6'^i^	155 (4)	O1—C1—C2	118.3 (9)
O6^i^—Cd1—O6'^i^	19.3 (9)	N1—C2—C3	108.8 (9)
O1^i^—Cd1—O6'^i^	78.7 (11)	N1—C2—C1	113.8 (9)
O1—Cd1—O6'^i^	75 (3)	C3—C2—C1	104.4 (9)
O5^i^—Cd1—O6'	86 (3)	N1—C2—H2	109.9
O5—Cd1—O6'	132 (3)	C3—C2—H2	109.9
O6—Cd1—O6'	19.3 (9)	C1—C2—H2	109.9
O6^i^—Cd1—O6'	155 (4)	C4—C3—C2	117.6 (10)
O1^i^—Cd1—O6'	75 (3)	C4—C3—H3A	107.9
O1—Cd1—O6'	78.7 (11)	C2—C3—H3A	107.9
O6'^i^—Cd1—O6'	142 (5)	C4—C3—H3B	107.9
O5^i^—Cd1—O2^i^	80.0 (4)	C2—C3—H3B	107.9
O5—Cd1—O2^i^	82.2 (4)	H3A—C3—H3B	107.2
O6—Cd1—O2^i^	93.9 (10)	C3—C4—C5	107.0 (13)
O6^i^—Cd1—O2^i^	87.2 (16)	C3—C4—C6	104.9 (12)
O1^i^—Cd1—O2^i^	54.4 (3)	C5—C4—C6	101.0 (15)
O1—Cd1—O2^i^	144.9 (3)	C3—C4—H4	114.2
O6'^i^—Cd1—O2^i^	100 (2)	C5—C4—H4	114.2
O6'—Cd1—O2^i^	86.3 (13)	C6—C4—H4	114.2
O5^i^—Cd1—O2	82.2 (4)	C4—C5—H5A	109.5
O5—Cd1—O2	80.0 (4)	C4—C5—H5B	109.5
O6—Cd1—O2	87.2 (16)	H5A—C5—H5B	109.5
O6^i^—Cd1—O2	93.9 (10)	C4—C5—H5C	109.5
O1^i^—Cd1—O2	144.9 (3)	H5A—C5—H5C	109.5
O1—Cd1—O2	54.4 (3)	H5B—C5—H5C	109.5
O6'^i^—Cd1—O2	86.3 (13)	C4—C6—H6A	109.5
O6'—Cd1—O2	100 (2)	C4—C6—H6B	109.5
O2^i^—Cd1—O2	160.6 (4)	H6A—C6—H6B	109.5
O5^i^—Cd1—C1^i^	104.8 (4)	C4—C6—H6C	109.5
O5—Cd1—C1^i^	107.1 (4)	H6A—C6—H6C	109.5
O6—Cd1—C1^i^	92.2 (18)	H6B—C6—H6C	109.5
O6^i^—Cd1—C1^i^	86.0 (9)	C12—C7—C8	120.0 (11)
O1^i^—Cd1—C1^i^	27.4 (3)	C12—C7—S1	121.3 (9)
O1—Cd1—C1^i^	117.9 (3)	C8—C7—S1	118.7 (9)
O6'^i^—Cd1—C1^i^	91.2 (11)	C9—C8—C7	120.4 (12)
O6'—Cd1—C1^i^	77 (2)	C9—C8—H8	119.8
O2^i^—Cd1—C1^i^	27.1 (3)	C7—C8—H8	119.8
O2—Cd1—C1^i^	172.3 (3)	C8—C9—C10	120.3 (12)
C2—N1—S1	120.3 (7)	C8—C9—H9	119.8
C2—N1—H1	107.3	C10—C9—H9	119.8
S1—N1—H1	106.5	C11—C10—C9	118.5 (12)
C1—O1—Cd1	92.5 (6)	C11—C10—H10	120.7
C1—O2—Cd1	89.0 (6)	C9—C10—H10	120.7
Cd1—O5—H5E	112.2	C10—C11—C12	122.3 (12)
Cd1—O5—H5F	111.9	C10—C11—H11	118.8
H5E—O5—H5F	109.8	C12—C11—H11	118.8
Cd1—O6—H6F	84.7	C7—C12—C11	118.4 (12)
H6E—O6—H6F	107.7	C7—C12—H12	120.8
Cd1—O6—H6'C	99.8	C11—C12—H12	120.8
			
O5^i^—Cd1—O1—C1	−40.1 (10)	Cd1—O1—C1—C2	−171.1 (9)
O5—Cd1—O1—C1	21.6 (9)	S1—N1—C2—C3	146.7 (7)
O6—Cd1—O1—C1	−96 (2)	S1—N1—C2—C1	−97.4 (9)
O6^i^—Cd1—O1—C1	88.2 (11)	O2—C1—C2—N1	158.6 (10)
O1^i^—Cd1—O1—C1	170.4 (8)	O1—C1—C2—N1	−22.2 (14)
O6'^i^—Cd1—O1—C1	92.4 (13)	O2—C1—C2—C3	−82.9 (13)
O6'—Cd1—O1—C1	−116 (3)	O1—C1—C2—C3	96.3 (12)
O2^i^—Cd1—O1—C1	178.1 (7)	N1—C2—C3—C4	−69.9 (14)
O2—Cd1—O1—C1	−4.2 (7)	C1—C2—C3—C4	168.2 (12)
C1^i^—Cd1—O1—C1	175.4 (4)	C2—C3—C4—C5	−68.8 (16)
O5^i^—Cd1—O2—C1	157.0 (9)	C2—C3—C4—C6	−175.5 (13)
O5—Cd1—O2—C1	−156.3 (9)	O3—S1—C7—C12	11.0 (11)
O6—Cd1—O2—C1	87 (2)	O4—S1—C7—C12	141.3 (10)
O6^i^—Cd1—O2—C1	−87 (2)	N1—S1—C7—C12	−103.7 (10)
O1^i^—Cd1—O2—C1	−5.3 (10)	O3—S1—C7—C8	−170.8 (9)
O1—Cd1—O2—C1	4.2 (7)	O4—S1—C7—C8	−40.5 (10)
O6'^i^—Cd1—O2—C1	−69 (3)	N1—S1—C7—C8	74.5 (10)
O6'—Cd1—O2—C1	72 (3)	C12—C7—C8—C9	−1.7 (19)
O2^i^—Cd1—O2—C1	−179.7 (7)	S1—C7—C8—C9	−179.9 (10)
C1^i^—Cd1—O2—C1	1(3)	C7—C8—C9—C10	0(2)
C2—N1—S1—O3	−44.3 (9)	C8—C9—C10—C11	0.4 (19)
C2—N1—S1—O4	−174.0 (8)	C9—C10—C11—C12	0(2)
C2—N1—S1—C7	70.9 (9)	C8—C7—C12—C11	2.2 (18)
Cd1—O2—C1—O1	−7.7 (12)	S1—C7—C12—C11	−179.6 (9)
Cd1—O2—C1—C2	171.4 (9)	C10—C11—C12—C7	−1.5 (19)
Cd1—O1—C1—O2	8.0 (13)		

Symmetry codes: (i) −*x*+1, −*y*+1, *z*.

### Hydrogen-bond geometry (Å, °)

**Table:**

*D*—H···*A*	*D*—H	H···*A*	*D*···*A*	*D*—H···*A*
N1—H1···O1	0.90	2.29	2.776 (11)	113
N1—H1···O2^ii^	0.90	2.35	3.121 (12)	143
O5—H5E···O1^iii^	0.85	1.85	2.639 (19)	154
O5—H5F···O1^iv^	0.85	1.79	2.605 (18)	161
O7—H7C···O3^v^	0.85	2.20	2.99 (2)	155
O7—H7D···O4^vi^	0.85	2.22	3.00 (2)	152
C2—H2···O3	0.98	2.46	2.903 (13)	107
C2—H2···O4^iii^	0.98	2.58	3.457 (13)	149
C12—H12···O3	0.93	2.52	2.871 (14)	102

Symmetry codes: (ii) *x*, *y*, *z*+1; (iii) *x*, *y*, *z*−1; (iv) −*x*+1, −*y*+1, *z*−1; (v) *x*+1/2, −*y*+3/2, −*z*+1; (vi) *x*+1/2, −*y*+3/2, −*z*+2.
